# Left atrial contraction strain and controlled preload alterations, a study in healthy individuals

**DOI:** 10.1186/s12947-022-00278-1

**Published:** 2022-03-30

**Authors:** Peter Gottfridsson, Roman A’Roch, Per Lindqvist, Lucy Law, Tomi Myberg, Magnus Hultin, Alexander A’Roch, Michael Haney

**Affiliations:** 1 Department of Surgical and Perioperative Sciences, Anesthesiology and Intensive Care Medicine, Umeå University, 901 85 Umeå, Sweden; 2 Department of Surgical and Perioperative Sciences, Clinical Physiology, Umeå University, Umeå, Sweden; 3 Anesthesiology and Intensive Care Medicine, Surgical and Perioperative Sciences, Umeå University (Sunderby sjukhus), Umeå, Sweden

**Keywords:** Left atrium, Contractile function, Preload, Echocardiography, Speckle tracking

## Abstract

**Background:**

In order to assess left atrial contractile function in disturbed circulatory conditions, it is necessary to have a clear understanding of how it behaves in a normal resting state with changes in loading conditions. However, currently the understanding of this relationship is incomplete. We hypothesize that in healthy individuals, left atrial contraction strain and its peak strain rate are increased or decreased by increasing or decreasing preload, respectively.

**Methods:**

Controlled maneuvers used to change preload included continuous positive airway pressure by mask (CPAP 20 cmH_2_O) for preload decrease, and passive leg raise (15 degrees angle) for preload increase. Cardiac ultrasound 4-chamber views of the left atria and left ventricle were acquired at baseline and during maneuver. Acquired images were post processed and analyzed offline. Comparisons were made using paired t-test and means with 95% confidence interval.

**Results:**

There were 38 participants, complete results were obtained from 23 in the CPAP maneuver and 27 in the passive leg raise maneuver. For the CPAP group, left atrial contraction strain was 11.6% (10.1 to 13.1) at baseline and 12.8% (11.0 to 14.6) during the maneuver (*p* = 0.16). Left atrial contraction peak strain rate was − 1.7 s^− 1^ (− 1.8 to − 1.5) at baseline and − 1.8 s^− 1^ (− 2.0 to − 1.6) during the maneuver (*p* = 0.29). For the passive leg raise-group, left atrial contraction strain was 10.1% (9.0 to 11.2) at baseline and 10.8% (9.4 to 12.3) during the maneuver (*p* = 0.28). Left atrial contraction peak strain rate was − 1.5 s^− 1^ (− 1.6 to − 1.4) at baseline and − 1.6 s^− 1^ (− 1.8 to − 1.5) during the maneuver (*p* = 0.29). Left atrial area, an indicator of preload, increased significantly during passive leg raise and decreased during CPAP.

**Conclusion:**

In healthy individuals, left atrial contraction strain and its peak strain rate seem to be preload-independent.

**Trial registration:**

The study was 2018-02-19 registered at clinicaltrials.gov (NCT03436030).

**Supplementary Information:**

The online version contains supplementary material available at 10.1186/s12947-022-00278-1.

## Background

The relationship between of cardiac chamber preload and systolic function can be important when assessing patients with circulatory insufficiency to optimize the therapy. When cardiac chambers are not sufficiently loaded, steps to increase preload of the heart, including intravenous fluid therapy, can lead to improvement of circulatory status [1]. In contrast, when the cardiac chambers are overloaded due to inadequate cardiac systolic function, then reduction of preload could be indicated. However, there are many patients where there is circulatory insufficiency, though without a clear indication of cardiac loading status. In these instances, assessment of atrial systolic performance in relation to pre-systolic loading, can be valuable [2].

In order to assess left atrial (LA) contractile function in disturbed circulatory conditions, it is necessary to have a clear understanding of how LA contractile function behaves in a normal resting state with changes in loading conditions. However, currently the understanding of this relationship is incomplete. With readily accessible, non-invasive imaging of cardiac structure and function using ultrasound, LA function can be routinely assessed at the bedside. Quantification for LA function can be achieved using 2-dimensional speckle tracking to determine global longitudinal LA strain during atrial contraction (LASct) and its peak strain rate (pLASRct) [3]. Observations regarding the relation of LA volume and pressure with LA contractile function, including in a heart failure cohort, have been presented [4]. Genovese et al. showed that LASct was reduced by a large preload reduction using a tilting maneuver [5]. In patients being anesthetized for cardiac surgery, LASct was unaffected by positive pressure ventilation and general anesthesia [6] and in renal failure patients both LASct and pLASRct was unaffected by a preload reduction after hemodialysis [7].

However, understanding of the relationship between LA volume, LA pressure loading and LA contractile function in regard to altered loading conditions is yet to be fully investigated.

We hypothesize that in healthy individuals, LASct and pLASRct are increased or decreased by increasing or decreasing preload, respectively. This study aims to investigate this relationship in healthy study participants using 2 controlled interventions, designed to alter preload. A passive leg raise (PLR) maneuver, intended to increase preload, and a continuous positive airway pressure (CPAP) maneuver, intended to decrease preload in the left atrium.

## Methods

The study was 2018-02-19 registered at clinicaltrials.gov (NCT03436030).

### Participants

With ethical approval from the Regional Board for Ethics, Research with Humans (Dnr 2017–327-31 M), and in compliance with the Declaration of Helsinki, and participant consent, healthy volunteers of both genders were recruited to participate in the study.

### Controlled preload alteration maneuvers and assessment

The first maneuver was a PLR designed to temporarily increase preload [8]. The participants started supine, or in the left lateral position to optimize ultrasound image quality, with their trunk at a semi recumbent angle of 15°. After 3 min in the semi recumbent position, a baseline transthoracic echocardiograph (Vivid 9, GE Healthcare, Horten, Norway) with apical 4 chamber views (which were focused on the left ventricle (LV), LA and standard doppler measurements) was recorded. The participants legs were then the raised passively to 15° and the trunk lowered and additional 15° (to supine) by tipping the whole bed. After 20 s of PLR, recording of the same views as mentioned above were done. The position was maintained until recording of the planned ultrasound assessments were completed, which took no more than 3 min.

The CPAP maneuver was performed using a Boussignac CPAP mask with 20 cm H_2_O airway pressure [9]. Participants were carefully instructed regarding how to perform the CPAP maneuver and were then allowed to practice before starting the protocol. Each participant was placed in the supine or left lateral position to optimize ultrasound image quality. After a 1-min resting period, the same abbreviated echocardiograph protocol as in the PLR maneuver was performed and recorded. Then a relaxed single CPAP inspiration with a passive inspiratory hold was maintained during image collection. Image acquisition commenced after 5 s of CPAP, and the above-described views were recorded before allowing participant spontaneous exhalation and recovery (at least 1 min). The procedure was repeated until all planned views and images were collected, which could require up to 5 repeated cycles of the maneuver, each with a 1-min recovery period in between. A continuous ECG was recorded, as well as continuous blood pressure and heart rate using a separate non-invasive device (Finapres, Finapres Medical Systems, Enschede, The Netherlands) throughout testing.

**Fig. 1:**
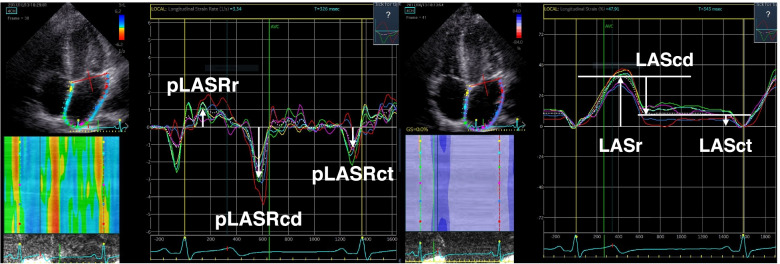
Representative example of strain assesment. Legend. A representative example of the left atrial strain and its peak strain rate measurement. Left atrial conduction strain (LAScd), peak strain rate conduction (pLASRcd), left atrial reservoir strain (LASr), peak strain rate reservoir (pLASRr), left atrial contraction strain (LASct), and peak strain rate contraction (pLASRct) are shown

Specific measurements using a commercially available software (Echo Pac 203 rev 66.4 GE Healthcare, Solna, Sweden) were performed offline to obtain the following parameters: 1. LASct and pLASRct, 2. LA reservoir strain (LASr) and peak reservoir strain rate (pLASRr) (a representative example is shown in Fig. 1), 3. LA area, 4. LV global longitudinal strain (LV GLS)/strain rate (LV GLSR), 5. LV outflow tract velocity time integral (LVOT VTI), 6. early trans mitral maximum flow velocity (E), 7. late trans mitral maximum flow velocity (A), 8. early diastolic maximal lateral mitral annular tissue velocity (e) and late diastolic maximal lateral mitral annular tissue velocity with atrial contraction (a), 9. maximal systolic lateral mitral annular tissue velocity (s), 10. heart rate (HR) from ECG, 11. LV end-diastolic volume was estimated from 4-chamber view using the Simpson’s method (LV EDV).

Echocardiographs were recorded and stored as DICOM files for offline analysis. At least 3 consecutive heart cycles were recorded for each measurement sequence.

### Analysis

LV and LA longitudinal strain and peak strain rate were measured in accordance with the standard published by EACVI/ASE/Industry Task Force with the zero-strain reference at end-diastole [3, 10]. The endocardial border was traced by the operator with a point-and-click method. Strain was measured from the assessor-chosen beat, based on timing of the intervention and optimally traceable endocardial borders.

### Statistical analysis

Primary outcomes of interest in this study were LASct and pLASRct, and they were analyzed in relation to the different preload conditions. Grouped results were tested for distribution normality using the Shapiro-Wilks test. Where non-normal distribution was observed, non-parametric statistical testing would be used. Grouped measures were presented with descriptive statistics (means and 95% confidence intervals) where appropriate. Paired comparisons were made using a paired t-test. A *p* value of less than 0.05 was considered statistically significant when assessing differences between pre-maneuver values and during maneuver values. Statistical analysis was performed using SPSS (IBM SPSS Statistics for Windows, Version 27.0. Armonk, NY, USA).

Based on our own pilot testing, a sample size estimation for paired T-testing was performed using a LASct estimate of 12 and 10% change with intervention, with standard deviation 2.5, alpha 0.05 and power 80% which gave an estimated sample size of 37.

## Results

**Fig. 2:**
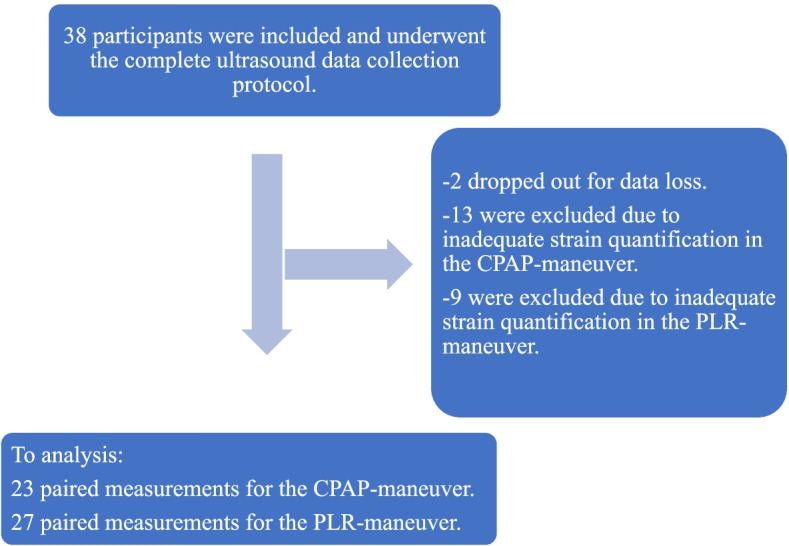
Participant flow chart. Legend: CPAP, continuous positive airway pressure; PLR, passive leg raise

Thirty-eight medical students were included, 18 females and 20 males. The mean age was 25.3 ± 3.6 years and mean BMI was 23.8 ± 1.9 kg/m^2^. All participants completed the interventions without symptoms. Data was lost for 2 participants when transferring from ultrasound machine to hard drive, resulting in 36 studies being included in the final analysis. These assessments were then assessed for imaging drop-out which was defined as inadequate atrial wall visualisation for 2-dimensional strain assessment. This resulted in 23 complete studies for the CPAP baseline and manoeuvre, and 27 studies for the PLR baseline and manoeuvre (Fig. 2, flow chart).

The primary result was that for both the CPAP and the PLR interventions, no change in LASct and pLASRct was found, even though atrial loading conditions were reduced or increased, respectively (Table 1).

**Table 1:** LASct and pLASRct pre- and during maneuvers

**CPAP** ***n*** **= 23**	**Pre CPAP**	**during CPAP**	***p*** **value**
LAS_ct_ (%)	11.6 (10.1 to 13.1)	12.8 (11.0 to 14.6)	0.157
pLASR_ct_ (s^−1^)	− 1.7 (− 1.8 to − 1.5)	− 1.8 (− 2.0 to − 1.6)	0.292
**PLR** ***n*** **= 27**	**Pre PLR**	**during PLR**	***p*** **value**
LAS_ct_ (%)	10.1 (9.0 to 11.2)	10.8 (9.4 to 12.3)	0.283
pLASR_ct_ (s^− 1^)	−1.5 (− 1.6 to − 1.4)	− 1.6 (− 1.8 to − 1.5)	0.233

Results presented as mean values with 95% confidence intervals. *CPAP* continuous positive airway pressure, *LASct* left atrial contraction strain, *pLASRct* peak left atrial contraction strain rate, *PLR* passive leg raise

### Secondary findings

The CPAP provocation led to changes indicative of lower atrial loading based on decreased LA area, LASr, HF, a, e, s, LVOT VTI (stroke volume), LV EDV, LV GLS (Table 2). The PLR provocation in the other hand led to changes indicative of higher atrial loading based on increased LA area, LASr, LVOT VTI (stroke volume).

**Table 2:** Secondary findings

**CPAP**	**Pre CPAP**	**CPAP**	***P*** **value**
LASr (%)	40.7 (38.3 to 43.1)	35.4 ± 33.1–37.6	< 0.001
pLASRr (s^− 1^)	1.8 (1.6 to 1.9)	1.5 (1.4 to 1.7)	0.018
LA Area (cm^− 2^)	16.0 (14.2 to 17.7)	14.1 (12.5 to 15.7)	0.026
LV GLS (%)	− 19.5 (− 20.4 to − 18.6)	− 18.2 (− 19.2 to − 17.1)	< 0.001
LV GLSR (s^− 1^)	− 1.0 (− 1.1 to − 0.9)	− 1.0 (− 1.1 to − 1.0)	0.319
LVOT VTI (cm)	23.8 (21.9 to 25.8)	18.4 (16.4 to 20.4)	< 0.001
E (m/s)	0.85 (0.80 to 0.91)	0.70 (0.66 to 0.74)	< 0.001
A (m/s)	0.43 (0.39 to 0.47)	0.40 (0.35 to 0.44)	0.085
e (m/s)	0.14 (0.13 to 0.15)	0,11 (0.10 to 0.13)	< 0.001
a (m/s)	0.057 (0.051 to 0.063)	0.051 (0.047 to 0.056)	0.009
s (m/s)	0.092 (0.083 to 0.10)	0.085 (0.076 to 0.094)	0.028
HR (min^− 1^)	60.3 (56.9 to 63.7)	57.0 (53.6 to 60.5)	0.055
LV EDV (ml)	107.0 (97.5 to 116.5)	90.6 (80.0 to 101.2)	0.002
**PLR**	**Pre PLR**	**PLR**	***P*** **value**
LASr (%)	37.4 (35.8 to 39.1)	41.5 (39.4 to 43.6)	0.001
pLASRr (s^− 1^)	1.48 (1.38 to 1.59)	1.69 (1.57 to 1.81)	0.013
LA Area (cm^2^)	14.9 (13.4 to 16.4)	16.4 (15.2 to 17.6)	0.007
LV GLS %	− 17.1 (− 17.8 to − 16.4)	−17.8 (− 18.8 to 16.8)	0.129
LV GLSR (s^− 1^)	− 0.98 (− 1.03 to − 0.94)	−0.96 (− 1.02 to − 0.89)	0.346
LVOT VTI (cm)	21.1 (19.7 to 22.5)	22.6 (21.4 to 23.7)	< 0.001
E (m)	0.79 (0.74 to 0.84)	0.79 (0.74 to 0.84)	0.907
A (m)	0.39 (0.36 to 0.41)	0.39 (0.35 to 0.42)	0.777
e (m)	0.14 (0.13 to 0.15)	0.15 (0.14 to 0.16)	0.124
a (m)	0.053 (0.048 to 0.058)	0.058 (0.053 to 0.063)	0.020
s (m)	0.089 (0.082 to 0.10)	0.094 (0.088 to 0.10)	0.046
HR (min^−1^)	59.3 (55.9 to 62.7)	59.3 (55.6 to 63.1)	0.962
LV EDV (ml)	103.8 (93.4 to 114.3)	106.3 (95.9 to 116.8)	0.384

Results presented as mean values with 95% confidence intervals. *A* late trans mitral maximum flow velocity, *a* late diastolic maximal lateral mitral annular tissue velocity with atrial contraction, *CPAP* continuous positive airway pressure, *E* early trans mitral maximum flow velocity, *e* early diastolic maximal lateral mitral annular tissue velocity, *HR* heart rate, *LA area* left atrial area, *LASct* left atrial contraction strain, *LV EDV* left ventricular end-diastolic volume, *LV GLS* left ventricular global longitudinal strain, *LV GLSR* left ventricular global longitudinal strain rate, *pLASRr* peak left atrial reservoir strain rate, *PLR* passive leg raise, *s* maximal systolic lateral mitral annular tissue

## Discussion

In this explorative study on healthy individuals, no clear change in LASct or pLASRct was found with these controlled loading and unloading maneuvers. The pre-systolic volume or loading indicators, including LA area and LASr, demonstrate that the model succeeded in bringing about load alterations.

There are few reports of LASct in cohorts during controlled load interventions. Genovese et al. who studied left atrium function by the use a preload decreasing model, a tilt test (designed for testing autonomic nervous system response). Their maneuver caused a large preload reduction by stepwise raising a tilting board from supine to an almost upright position. This decreased atrial preload substantially and also left atrial contraction strain. However there was activation of the autonomic nerve system demonstrated by an increase in heart rate at the upright position [5]. In one report, an increase in LASct was observed in response to leg lift in a hypertensive group but not in a comparison group with diastolic dysfunction and preserved ejection fraction [11]. In our study model for preload decrease (CPAP-maneuver), there was a small but measureable preload reduction which was not was associated with a clear change in LASct or pLASRct. Also, heart rate was not affected. Additionally, different interventions may lead to different degrees of activation of the autonomic nervous system. For example, the tilt test may activate the sympathetic nervous system more than our CPAP-maneuver.

LASct with presumed preload reductions has been assessed in clinical settings. In patients being anesthetized for cardiac surgery, LASct was unaffected by positive pressure ventilation and general anesthesia [6], though the preload changes were occurring in the midst of a complex circulatory intervention. LASct and pLASRct has been described in renal failure patients, before and after hemodialysis and was apparently unaffected by dialysis and preload reduction [7].

### The model’s validity

These preload interventions in healthy young individuals were successful and reflect generally normal physiology. The intent was to bring about preload changes without large simultaneous changes in sympathetic/parasympathetic nervous system balance (as demonstrated by heart rate), related to atrial contractility.

There were clear changes in preload, as shown by LA area and LVOT VTI [12]. The baseline values obtained in this study agree well with reported atrial strain values for young and healthy individuals [13]. It is possible that, with aging and cardiovascular disease, atrial contractile functional responses to loading alterations can differ, however this was not investigated in this study.

Paired comparisons for each participant allowed assessments of intervention effects to be performed with a relatively small cohort. Still, this study was not powered to detect small changes in LASct.

### Secondary findings

The load alteration during CPAP interventions has been shown to be associated with a small change in LV GLS with simultaneous change in LAS during the same cardiac cycle [14]. Our findings confirm this interaction (Table 2).

At the same time, our findings show that during maneuvers there were no changes in LV GLSR values despite changes in LA SRr. This type of finding in the clinical setting might be explained by differences related to thick-walled LV and thin-walled LA, where a less compliant ventricle presumable affects atrial systole.

### Limitations of the study design

The model included gentle manipulations of atrial preload in healthy young participants. These findings need to be followed up in a middle-aged cohort, where clinical questions are more common. Rest periods were adequate as demonstrated by no difference in the starting heart rates for each intervention. Measurement sequences were standardized, but there was still a possible influence of compounding maneuver effect due to repetition needed for all the ultrasound recording sequences.

Baseline assessments for the two interventions were taken with slightly different postures, where the PLR maneuver started with semi-recumbent position and CPAP maneuver were lying flat. The grouped baseline means for the two manoeuvres are thereby not directly comparable. More forceful interventions could have provoked larger transient changes in cardiac chamber loading. However, this may have risked for activating immediate sympathetic nerve system responses which would have complicated analysis of atrial performance and increased bias. Atrial afterload is important when the ventricle is overloaded and straining. However, in this experimental setting it could be assumed that the ventricle was minimally loaded and normally compliant as participants were young and healthy. The technical aspects of atrial strain assessment present challenges concerning adequate image quality, where this could vary within the same sequence. Careful echocardiographic measurement is needed to generate reliable assessments with this type of outcome. The assessor doing the strain measurements was not blinded to the interventions, since assessment of the whole sequence was necessary in order to get the optimal heart cycles.

The study cohort was small, and therefore potentially not adequate to detect possible small true effects of the interventions given the usual within-group variability with this type of ultrasound measure.

## Conclusions

In healthy individuals, LASct and pLASRct seem to be preload independent. The LA strain as a measure of intrinsic LA function and its load dependence/independence in different cardiac disease settings needs to be further evaluated.

## Supplementary Information

**Additional file 1.** Individual level data and summary values, analysis dataset.

## Data Availability

Individual level data is included as a supplementary file.

## Abbreviations

A: Late trans mitral maximum flow velocity
a: Late diastolic maximal lateral mitral annular tissue velocity with atrial contraction
CPAP: Continuous positive airway pressure
E: Early trans mitral maximum flow velocity
e: Early diastolic maximal lateral mitral annular tissue velocity
HR: Heart rate
LA: Left atrial
LASct: Left atrial contraction strain
LV: Left ventricle
LV EDV: Left ventricular end-diastolic volume
LV GLS: Left ventricular global longitudinal strain
LV GLSR: Left ventricular global longitudinal strain rate
pLASRct: Peak left atrial contraction strain rate
pLASRr: Peak left atrial reservoir strain rate
PLR: Passive leg raise
s: Maximal systolic lateral mitral annular tissue
