# Correction to: Left atrial contraction strain and controlled preload alterations, a study in healthy individuals

**DOI:** 10.1186/s12947-022-00281-6

**Published:** 2022-04-26

**Authors:** Peter Gottfridsson, Roman A’Roch, Per Lindqvist, Lucy Law, Tomi Myrberg, Magnus Hultin, Alexander A’Roch, Michael Haney

**Affiliations:** 1 Department of Surgical and Perioperative Sciences, Anesthesiology and Intensive Care Medicine, Umeå University, 901 85 Umeå, Sweden; 2 Department of Surgical and Perioperative Sciences, Clinical Physiology, Umeå University, Umeå, Sweden; 3 Anesthesiology and Intensive Care Medicine, Surgical and Perioperative Sciences, Umeå University (Sunderby sjukhus), Umeå, Sweden

**Correction to: Cardiovasc Ultrasound 20, 8 (2022)**

**https://doi.org/10.1186/s12947-022-00278-1**

Following the publication of the original article [1], the author reported that one of the co-authors name was misspelled. “Tomi Myberg“ should be “Tomi Myrberg“.

The original article has been updated.
